# Epigenetic histone modulation contributes to improvements in inflammatory bowel disease via EBI3

**DOI:** 10.1007/s00018-020-03451-9

**Published:** 2020-01-18

**Authors:** Alexandra Wetzel, Bettina Scholtka, Christian Gerecke, Burkhard Kleuser

**Affiliations:** Department of Nutritional Toxicology, Institute of Nutritional Science, University of Potsdam, Arthur-Scheunert-Allee 114-116, 14558 Nuthetal, Germany

**Keywords:** Histone deacetylase inhibitor, Inhibitory cytokines, Interleukin-35, SAHA, Ulcerative colitis

## Abstract

Ulcerative colitis (UC) is characterized by relapsing–remitting inflammatory episodes paralleled by varying cytokine levels, suggesting that switching epigenetic processes might be involved. However, the epigenetic impact on cytokine levels in colitis is mostly unexplored. The heterodimeric interleukin (IL)-12 cytokine family have various functions in both pro- and anti-inflammatory processes. The family member IL-35 (EBI3/IL-12p35) was recently reported to play an anti-inflammatory role in UC. Therefore, we aimed to investigate a possible epigenetic regulation of the IL-35 subunits in vitro and in vivo, and to examine the epigenetic targeting of EBI3 expression as a therapeutic option for UC. Exposure to either the pro-inflammatory TNFα or to histone deacetylase inhibitors (HDACi) significantly increased *EBI3* expression in Human Colon Epithelial Cells (HCEC) generated from healthy tissue. When applied in combination, a drastic upregulation of *EBI3* expression occurred, suggesting a synergistic mechanism. Consequently, IL-35 was increased as well. In vivo, the intestines of HDACi-treated wild-type mice exhibited reduced pathological signs of colitis compared to non-treated colitic mice. However, the improvement by HDACi treatment was completely lost in Ebi3-deficient mice (*Ebi3*^*−/−*^). In fact, HDACi appeared to exacerbate the disease phenotype in *Ebi3*^−/−^. In conclusion, our results reveal that under inflammatory conditions, *EBI3* is upregulated by the epigenetic mechanism of histone acetylation. The in vivo data show that the deficiency of *EBI3* plays a key role in colitis manifestation. Concordantly, our data suggest that conditions promoting histone acetylation, such as upon HDACi application, improve colitis by a mechanism involving the local formation of the anti-inflammatory cytokine IL-35.

**Electronic supplementary material:**

The online version of this article (10.1007/s00018-020-03451-9) contains supplementary material, which is available to authorized users.

## Introduction

Ulcerative colitis (UC) and Crohn’s disease represent the most frequent chronic inflammatory diseases of the gastrointestinal tract. They present with a physically demanding clinical course and are associated with an increased risk of colorectal cancer [1–3]. The pathogenic mechanisms associated with their advancing inflammation are still not completely understood, though intestinal dysbiosis, disturbance in epithelial barrier function, and imbalances within the immune system are known to play roles [4]. Intracellularly, the inflammatory process is accompanied by oxidative stress-induced gene mutations that impair cellular repair mechanisms and apoptosis [5]. Epigenetic alterations are also known to accompany their disease progressions [6].

Under inflammatory conditions, affected tissues are infiltrated by immune cells secreting a range of pro-inflammatory [e.g., Interleukin (IL)-1, IL-6, and TNFα] and anti-inflammatory (TGF-β1, IL-4, and IL-10) cytokines. Among these, the IL-12 family consists of dimeric pro- as well as anti-inflammatory cytokines that play important roles in lymphocyte differentiation. Unusually, the alpha-subunits of IL-12 family cytokines are interchangeable and can be combined with beta-subunits to form various heterodimers. The same applies to the corresponding receptors. One member of the IL-12 family, IL-35, has been linked with colitis as an anti-inflammatory cytokine [7, 8]. IL-35 is a dimer consisting of the Epstein–Barr virus-induced gene 3 (EBI3) and IL-12p35 subunits [9]. While IL-12p35 also dimerizes with IL-12p40 to form the pro-inflammatory cytokine IL-12, EBI3 can dimerize with IL-27p28 to form the anti-inflammatory IL-27 [10], or with IL-23p19 [11] to form IL-39 [12].

The EBI3 glycoprotein shares 27% protein homology with IL-12p40 [13] and promotes lymphocyte activation during inflammatory processes. In particular, differentiation of helper T 1 (T_H_1), T_H_17, and regulatory T (Treg) cells are induced involving signal transducer and activator of transcription (STAT) 3 signaling [14]. Through use of *Ebi3-*deficient (*Ebi3*^*−/−*^) mice, it has also been demonstrated to affect T_H_2-type immune responses via invariant natural killer T cells [15]. *EBI3* was shown to be a nuclear factor ‘kappa-light-chain-enhancer’ of activated B-cell (NFκB) target gene in human intestinal endothelial cells, linking its upregulation to the activity of tumor necrosis factor α (TNFα), a pro-inflammatory cytokine capable of inducing NFκB nuclear translocation and the induction of further pro- and anti-inflammatory cytokines [16]. Using *IL-27p28* and *EBI3* knockout mice, it was shown that of the two anti-inflammatory cytokines, IL-35 rather than IL-27 controlled the development of T-cell-dependent colitis [8]. Concordantly, stimulation of peripheral blood mononuclear cells from UC patients with IL-35, but not IL-27, was able to induce IL-10 [17]. IL-10 is another immunomodulatory cytokine with a central function in suppression of excessive inflammation finding expression in the fact that *IL-10*-deficient mice spontaneously develop colitis [18].

Histone deacetylase inhibitors (HDACi) have previously been shown to have a positive impact upon the course of colitis [19–21]. Aggravating and suppressing conditions during relapsing phases of colitis led us to the assumption that the switch function of epigenetic processes might also be involved in differential cytokine regulation during inflammatory diseases like colitis. Interestingly, TNFα and connected gene networks were evidently elicited by the HDACi Trichostatin A (TSA) in cardiac myocytes [22]. Furthermore, the duration of NFκB action is known to be regulated by various HDACs [23]. To investigate whether histone modifications play a role in regulating the expression of *EBI3* and its binding partners, we employed Human Colon Epithelial Cells (HCEC), generated from healthy colon instead of fully developed neoplasias. In addition to this, we induced a chronic colitis model in *Ebi3*^−/−^ mice to examine the epigenetic targeting of *EBI3* expression as a possible therapeutic option. Here, we demonstrate that under inflammatory conditions *EBI3* is upregulated by the epigenetic mechanism histone acetylation. Our data reveal that *EBI3* as the greater inducible subunit of the anti-inflammatory acting IL-35 has a large impact on the HDACi-induced improvement of colitis symptoms.

## Materials and methods

### Cell culture and treatment

HCEC (Human Colon Epithelial Cells) were obtained from Nestlé Ltd. Research Center (Lausanne, Switzerland). Cells were cultured in Dulbecco’s Modified Eagle’s Medium (DMEM) with 2 mM L-glutamine supplemented with 10% fetal bovine serum, 45 IU/ml penicillin, 45 IU/ml streptomycin, 9.11 µl/ml sodium pyruvate, 4.9 × 10^–3^ µl/ml phosphoethanolamine, 4.9 × 10^–3^ µl/ml ethanolamine, and 3 mg/ml BSA until 90–95% confluency before being subcultivated. For each experiment, 1 × 10^6^ cells were seeded on 150 mm diameter dishes. After 24 h, when cells were in the exponential phase of growth, they were stimulated with 1 µM Vorinostat (SAHA, Sigma-Aldrich, Steinheim, Germany), or 30 ng/ml (~ 0,1 µM) Trichostatin A (TSA, Sigma-Aldrich, Steinheim, Germany) for the indicated time period. For the last 24 h of the incubation TNFα (20 ng/ml) (Miltenyi Biotec GmbH, Bergisch Gladbach, Germany) or vehicle was added. Vehicle controls were performed for every time point. Cultivation of cells was carried out in a humidified incubator at 37 °C with 5% CO_2_ in O_2_. Before use, the cells were tested for mycoplasma contamination.

### MTT assay

To determine possible cytotoxic effects induced by the stimulants a 3-(4,5-dimethylthiazol-2-yl)-2,5-diphenyltetrazolium bromide (MTT, Sigma-Aldrich, Steinheim, Germany) reduction assay (MTT assay) was used [24]. HCEC cells were seeded into 96-well plates (TPP, Trasadingen, Switzerland) (8000 cells per well). The cells were treated for 48 and 72 h with different concentrations of TSA or SAHA 24 h after seeding. For the parallel stimulation with TSA or SAHA, TNFα was added for the last 24 h. The positive controls were treated with 0.02% SDS. Untreated controls were incubated for the same duration. After the incubation time, the cells were washed with PBS and treated with 100 µl MTT solution per well (0.5 mg/ml) for 4 h at 37 °C. Subsequently, the supernatants were removed and 50 µl dimethyl sulfoxide (DMSO, Carl Roth, Karlsruhe, Germany) were added. To dissolve the formazan salt, the plates were shaken at 300 rpm for 10 min at room temperature. The optical density at 540 nm was measured using a microplate reader (Tecan, Crailsheim, Germany). A cell viability < 75% predicts cytotoxic effects.

### Analysis of gene expression

Total RNA from cells was extracted with the High Pure RNA Isolation Kit (Roche, Mannheim, Germany). RNA extraction from tissue was performed with RNeasy Mini Kit (Qiagen, Hilden, Germany). RNA concentration and purity were determined using a NanoVue^™^ Plus UV–Vis spectrophotometer (GE Healthcare, Berlin, Germany). Only RNA with the ratio 2.0 of absorbance at 260/280 nm was used. The isolated mRNA was reverse-transcribed using the RevertAid reverse transcriptase (Thermo Fisher, Darmstadt, Germany, according to the protocol). The quantitative Reverse Transcription-PCR (RT-qPCR) was performed using the Maxima SYBR Green qPCR Mix (ThermoFisher, Darmstadt, Germany) on a LightCycler 480 II Real-Time PCR system (Roche, Mannheim, Germany). Quantification was done with the ΔΔ Ct method [25] with *h*-*HMBS* and *m*-*Hprt* served as reference genes. The oligonucleotide primers are listed in Table 1 (human) and Table 2 (murine). All primers were tested with positive controls by performing melting profiles following RT-qPCR and product sizes were checked by agarose gel electrophoresis. PCR conditions were as follows: 42 cycles of 15 s at 95 °C, 15 s at annealing temperature (60 °C for *h-IL-12p40* and *h-IL-23p19*, and 58 °C for all other genes), and 15 s at 72 °C. Specimens were assayed in duplicates of at least three independent experiments as indicated.

**Table 1:** Oligonucleotide primer sequences (human) for gene expression analysis by RT-qPCR and expected product sizes

Target gene	Gene accession number	Sequence	Product size (bp)
*h-HMBS*	NM_000190.3	fw: ACCAAGGAGCTTGAACATGC rv: GAAAGACAACAGCATCATGAG	143
*h-EBI3*	NM_005755.2	fw: ATTGCCACGTACAGGCTCGG rv: ACATTGAGCACGTAGGGAGC	131
*h-IL-12p35*	NM_000882.3	fw: ACAGTGGAGGCCTGTTTACC rv: ACTCCCATTAGTTATGAAAGAGGTC	87
*h-IL-27p28*	*NM_145659.3*	fw: CAGGCGACCTTGGCTGG rv: CAGGTGAGATTCCGCAAAGC	206
*h-IL-12p40*	*NM_002187.2*	fw: GCCCAGAGCAAGATGTGTCA rv: CACCATTTCTCCAGGGGCAT	150
*h-IL-23p19*	NM_016584.2	fw: AGGCAAAAAGATGCTGGGGA rv: TCCTTTGCAAGCAGAACTGAC	287

The italic indicated gene accession numbers for the analyzed genes refer to the GenBank^®^ sequence database provided by the National Center for Biotechnology Information (NCBI, USA)

*HMBS*, hydroxymethylbilane synthase, *EBI3* Epstein–Barr virus-induced gene 3, *IL* interleukin, *fw* forward, *rv* reverse, *bp* base pairs

**Table 2:** Oligonucleotide primer sequences (murine) for gene expression analysis by RT-qPCR and expected product sizes

Target gene	Gene accession number	Sequence	Product size (bp)
*m-Hprt*	NM_013556.2	fw: TGGATACAGGCCAGACTTTGTT rv: CAGATTCAACTTGCGCTCATC	162
*m-Tnfα*	NM_001278601.1	fw: GGCAGGTCTACTTTGGAGTC rv: ACATTCGAGGCTCCAGTGAATTCGG	300
*m-Ikbkb*	NM_010546.2	fw: CGGCCCTTCCTCCCTAAC rv: GGTGCCACATAAGCATCAGC	196
*m-Nfkb1*	XM_006501106.3	fw: ACACGAGGCTACAACTCTGC rv: TCCCGGAGTTCATCTCATAGT	162
*m-Il-17a*	NM_010552.3	fw: TCAAAGCTCAGCGTGTCCAA rv: TCTTCATTGCGGTGGAGAGTC	162
*m-Bcl-2*	NM_009741.5	fw: GGATAACGGAGGCTGGGATGC rv: ACTTGTGGCCCAGGTATGC	149
*m-Bcl-xL*	NM_001355053.1	fw: CGGCTGGGACACTTTTGTGG rv: CTGGTAGCAATGGTGGCTGA	226
*m-Ifnγ*	NM_008337.4	fw: AGGAACTGGCAAAAGGATGGT rv: TCATTGAATGCTTGGCGCTG	236
*m-Il-10*	NM_010548.2	fw: ACTACCAAAGCCACAAGGCA rv: TGGCAACCCAAGTAACCCTTA	287
*m-Il-6*	NM_031168.2	fw: TGGAGTCACAGAAGGAGTGGCTAAG rv: TCTGACCACAGTGAGGAATGTCCAC	155

The indicated gene accession numbers for the analyzed genes refer to the GenBank^®^ sequence database provided by the National Center for Biotechnology Information (NCBI, USA)

*Bcl-2* B-cell lymphoma-2, *Bcl-xL* B-cell lymphoma-extra large, *Hprt* hypoxanthine–guanine phosphoribosyltransferase, *Ifnγ* Interferon gamma, *Ikbkb* inhibitor of nuclear factor Kappa B kinase subunit beta, *IL* interleukin, *NF-κB* nuclear factor 'kappa-light-chain-enhancer' of activated B-cells, *Tnfα* tumor necrosis factor alpha, *fw* forward, *rv* reverse, *bp* base pairs

### Immunoblotting

For protein concentration, cell culture supernatants were incubated on ice for 15 min with 10% trichloroacetic acid followed by centrifugation (12,000×*g*, 5 min, 4 °C). The resulting pellet was washed with ice-cold acetone and then resuspended with PBS. After neutralizing with 1 M Tris, the samples were boiled in SDS sample buffer and were analyzed by immunoblotting. Proteins were separated by 12% SDS-polyacrylamide gel electrophoresis (SDS-PAGE) and blotted onto PVDF membranes. After blocking with 5% non-fat dry milk in TBST, the membranes were incubated with the primary antibody anti-EBI3 (EPR5747 (ab124694), Abcam, Cambridge, UK) overnight at 4 °C, followed by incubation with secondary anti-rabbit IgG HRP linked antibody (#7074, Cell Signaling Technology, Frankfurt, Germany) for 1 h at room temperature. Detection was performed with Clarity Western ECL Substrate according to the manufacturer’s protocol using a ChemiDoc XRS + system (Bio-Rad Laboratories, Munich, Germany). To prove that the secreted proteins came from the same number of attached cells, the corresponding cells were lysed in RIPA buffer and the total protein was determined by Bradford assay [26]. For visualization, the same volumes of cell lysates were analyzed by immunoblotting as above with an antibody against β-Actin (ab8226 Abcam, Cambridge, UK).

### Mice

Mice deficient for EBI3 (*Ebi3*^*−/−*^) were purchased from Jackson Laboratory (USA). *Ebi3*^*−/−*^ mice and their corresponding wild-type C57BL/6 were bred and maintained in microisolator cages under specific pathogen-free conditions. All animals used in experiments were genotyped according to the instructions provided by Jackson Laboratory. The animals had free access to food and water, and were kept with a diurnal 12 h light and dark cycle in accordance with national guidelines. Animal experiments were performed with each fifteen 6–8-week-old male mice per group. At the end of the experiment, mice were sacrificed by isoflurane anesthesia and subsequent blood collection by cardiopuncture. Experiments were approved by the responsible authorities (LUGV Brandenburg, Germany; 2347-30-2017).

### Induction of chronic dextran sulfate sodium (DSS)-induced colitis

Chronic colitis was induced by cyclic DSS (MP Biomedicals Germany GmbH, Eschwege) administration in drinking water. *Ebi3*^*−/−*^ as well as C57BL/6 were exposed to three cycles consisting of 1.5% DSS for 7 days followed by water without DSS for 14 days. The total duration of the experiment was 9 weeks.

### Clinical assessment of colitis

For the clinical evaluation of colitis, body weight loss and the stool consistency (1 point for soft but shaped stool, 2 points for irregular formed feces, 3 points for more liquid feces, 4 points for severe diarrhea; decided per cage) were determined as described elsewhere [27]. At the end of the experiment, the entire colon was removed from the cecum to the anus. Colon length and weight were measured as a marker of colitis. As a further inflammatory marker, the spleen weight was determined.

### HDACi treatment of colitic mice

After each week of DSS administration, *Ebi3*^*−/−*^ as well as C57BL/6 were treated daily for 5 days with 25 mg/kg body weight Vorinostat (SAHA, Cayman Chemical, Michigan, USA). SAHA was dissolved in 10% DMSO/phosphate-buffered saline. HDAC inhibitor solution and vehicle control were administered intraperitoneally (i.p.).

### Immunohistochemical analysis of colitis

For histological evaluation of colitis, cross sections of the formalin-fixed and paraffin-embedded colon were prepared. Immunohistochemistry was performed with antibodies against CD3 (anti-CD3, New England Biolabs, Frankfurt, Germany), a T-cell marker, and Caspase-3 (anti-Caspase-3, Dako Deutschland GmbH, Hamburg, Germany) as a marker of apoptosis.

### Statistical analysis

Data are presented as means ± standard error of the mean (SEM). If *n* < 5, the individual data points from each experiment were plotted. Statistical analysis was performed using the log-rank test for survival analysis, unpaired Student *t* test for comparison of two groups, and one-way ANOVA with Sidak’s post hoc test or two-way ANOVA with Tukey’s post hoc test as indicated for more than two data sets (*p < 0.05; **p < 0.01; ***p < 0.001; ****p < 0.001) with the software GraphPad Prism (GraphPad Software, Inc., La Jolla, USA).

## Results

### Epigenetic modification by HDAC inhibition drastically increases TNFα-induced *EBI3* expression

**Fig. 1:**
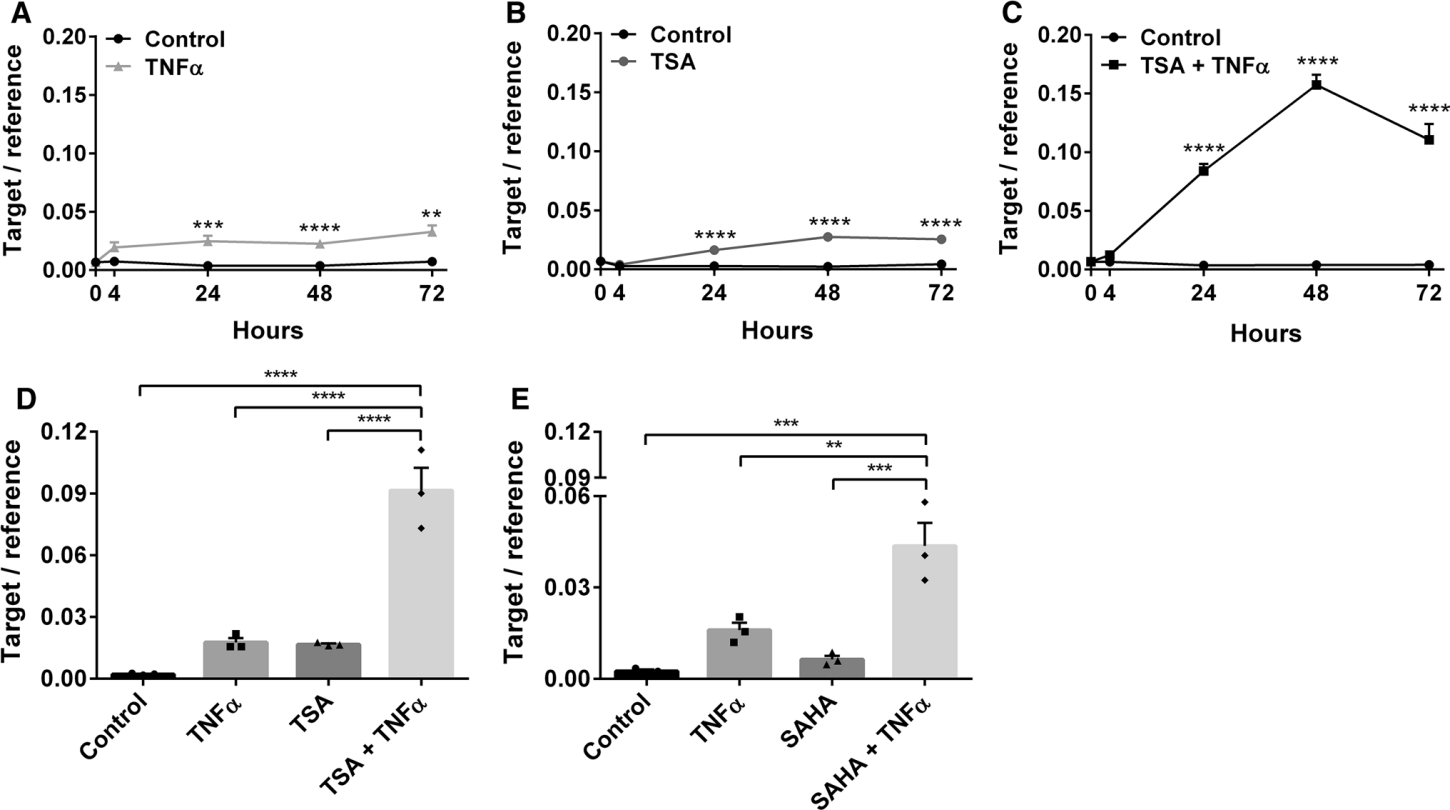
HDACi and TNFα induce a synergistic increase in *EBI3* mRNA expression. HCEC cells were stimulated for up to 72 h with TNFα, TSA or both substances. Subsequently, the *EBI3* mRNA expression was determined by RT-qPCR and referred to *HMBS* as housekeeping gene. **a–c** the *EBI3* mRNA expression was time-dependently increased in HCEC by TNFα (**a**), TSA (**b**), or TSA together with TNFα (**c**). Data were normalized to the reference gene and compared to vehicle-stimulated cells by two-way ANOVA and Sidak’s post hoc test. The graphs show the mean ± SEM from three independent experiments (**p < 0.01; ***p < 0.001; ****p < 0.0001). **d, e** To determine the combinatorial effect of inflammatory stimulus and HDACi, HCEC were stimulated with TSA (**d**) or SAHA (**e**) for 48 h and were treated or not with TNFα. The *EBI3* mRNA expression was determined as in **a**–**c**. Data are presented as mean ± SEM from three independent experiments. Statistical analysis was performed using one-way ANOVA and Tukey’s post hoc test (**p < 0.01; ***p < 0.001; ****p < 0.0001)

Previous studies in diverse cancer cell lines have shown that *EBI3* expression can be induced by TNFα stimulation via NFκB promoter activity [28, 29]. Here, we aimed to determine whether this same phenomenon occurs in HCEC, generated from healthy colon. TNFα stimulation of HCEC significantly increased basal *EBI3* mRNA levels from 24 h onwards (6.5-fold as determined by RT-qPCR) (Fig. 1a), but the expression level was relatively low compared to the expression of the housekeeper gene *HMBS*. HCEC were then treated with TSA, a prototype HDACi commonly used in cell culture experiments. TSA stimulation resulted in a significant enhancement in *EBI3* expression from 24 h onwards, peaking with a ninefold increase at 48 h (Fig. 1b). TSA induced *EBI3* expression in a dose-dependent manner (Supplementary Fig. S1a). As HDACi treatment of HCEC should increase the accessibility of the *EBI3* gene, we hypothesized that combinatorial TNFα and TSA treatments could lead to further upregulation of *EBI3*. Indeed, costimulation with TSA and TNFα resulted in an unexpected drastic increase in *EBI3* expression compared to either treatment alone, peaking at a 41-fold increase relative to the vehicle control after 48 h (Fig. 1c, d). Therefore, under non-inflammatory conditions *EBI3* is expressed at a very low level and increases after pro-inflammatory cytokine TNFα stimulation. However, an additional epigenetic regulation drastically increased *EBI3* formation. This effect was confirmed using SAHA, another HDACi with more clinical relevance. Stimulation of HCEC with SAHA resulted in a 2.5-fold increase of *EBI3* mRNA expression, while concurrent stimulation with SAHA and TNFα synergistically increased *EBI3* levels by 18-fold (Fig. 1e). The *EBI3*-inducing effect of SAHA was also dose-dependent (Supplementary Fig. S1b). The applied doses of the stimulatory agents were proven to be not cytotoxic nor proliferative by means of MTT assays (Supplementary Fig. S2).

### EBI3 protein formation and secretion is enhanced upon HDAC inhibition and TNFα application

**Fig. 2:**
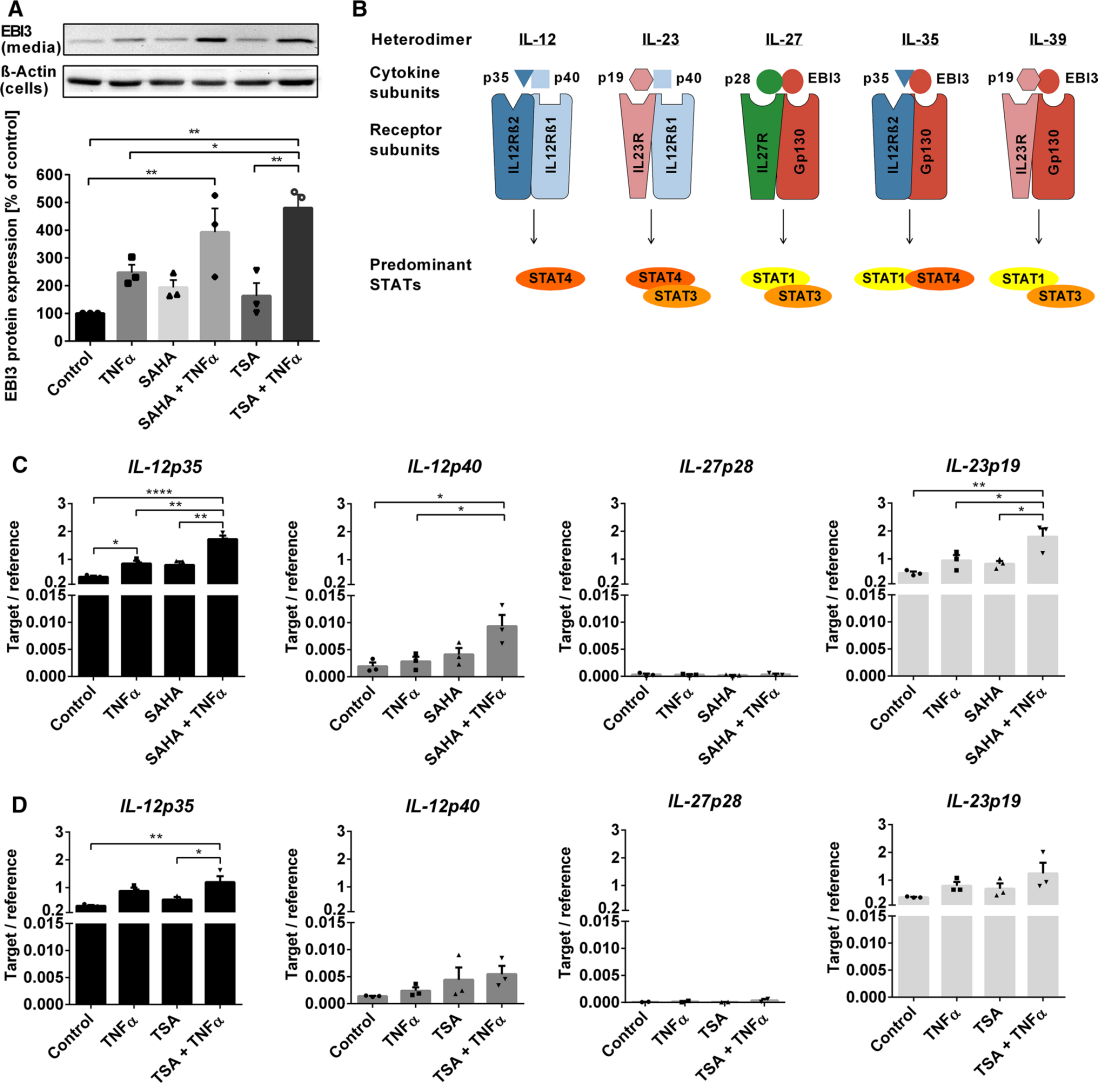
HDACi and TNFα stimulation enhance EBI3 protein secretion and differentially regulates gene expression of IL-12 cytokine family subunits. HCEC cells were stimulated with SAHA or TSA for 48 h and were treated or not with TNFα. **a** The proteins in the cell culture supernatants were precipitated with trichloroacetic acid, separated by SDS-PAGE, and blotted onto PVDF membranes. EBI3 was detected with anti-EBI3 antibody. The differences in EBI3 protein levels were statistically analyzed by one-way ANOVA and Tukey’s post hoc test. The graph shows the mean ± SEM from three independent experiments (*p < 0.05; **p < 0.01). To visualize that the supernatant came from the same number of cells, the same volumes of whole cell lysates were analyzed by immunoblotting with anti-β-Actin antibody. **b** The diagram gives an overview of the IL-12 cytokine family members, their subunits, receptors, and predominant STATs. **c, d** The expression profile of the IL-12 cytokine subunits is differentially regulated by HDACi and TNFα. HCEC were stimulated with SAHA (**c**) or TSA (**d**) for 48 h and treated or not with TNFα. The mRNA expression of *IL-12p35*, *IL-12p40*, *IL-27p28,* and *IL-23p19* was determined by RT-qPCR and referred to *HMBS* as housekeeping gene. For effective comparison of expression data, the y-axes are consistently formatted. Statistical analysis was performed using one-way ANOVA and Tukey’s post hoc test. Data are shown as mean ± SEM from three independent experiments (*p < 0.05; **p < 0.01; ****p < 0.0001)

In order to confirm the stimulatory effect by HDACi and TNFα on the protein level, HCEC were treated with TNFα together with SAHA or TSA. Since EBI3 has to be secreted to exert its action by binding to specific cell surface receptors, its protein expression was assessed by Western blot of protein precipitated from media of stimulated HCEC. TNFα treatment led to an increase in secreted EBI3 as compared to the vehicle control. As expected, combinatorial treatments with TNFα and either SAHA or TSA resulted in markedly higher EBI3 protein secretion (fourfold and fivefold, respectively) (Fig. 2a).

### HCEC express high levels of the EBI3-binding partners *IL-12p35* and *IL-23p19*

Since EBI3 is able to dimerize with different members of the IL-12 cytokine family (Fig. 2b), the mRNA expression of these subunits in HCEC was determined after stimulation with the HDACi alone or in combination with TNFα. Two further subunits of the IL-12 cytokine family, *IL-12p35* and *IL-23p19,* were constitutively expressed at a high-level relative to the low constitutive expression of *EBI3* in unstimulated HCEC (Fig. 2c, d). Simultaneous treatment of HDACi and TNFα led to a three-to-fourfold increase in both subunits with either SAHA (Fig. 2c) or TSA (Fig. 2d). These were also epigenetically regulated, but to a lesser extent than seen with *EBI3. IL-27p28* expression was almost absent in HCEC. IL-12p40 could compete for EBI3-binding partners IL-12p35 or IL-23p19. Combined HDACi and TNFα stimulation resulted in a four-to-fivefold increase in *IL-12p40* (Fig. 2c, d). However, *IL-12p40* expression was still very low. In summary, in the course of an inflammatory process based on TNFα signaling, the production of *EBI3* and consequently of the dimeric IL-12 family cytokines containing the EBI3 gene product, the anti-inflammatory IL-35 or the pro-inflammatory IL-39, could be further increased by histone deacetylase inhibition in human colon epithelial cells. Direct measurement of the dimeric IL-35 or IL-39 proteins was not possible due to a lack of discriminating antibodies against the subunits.

### *Ebi3*^*−/−*^ mice show a more pronounced chronic DSS-induced colitis phenotype than wild-type

**Fig. 3:**
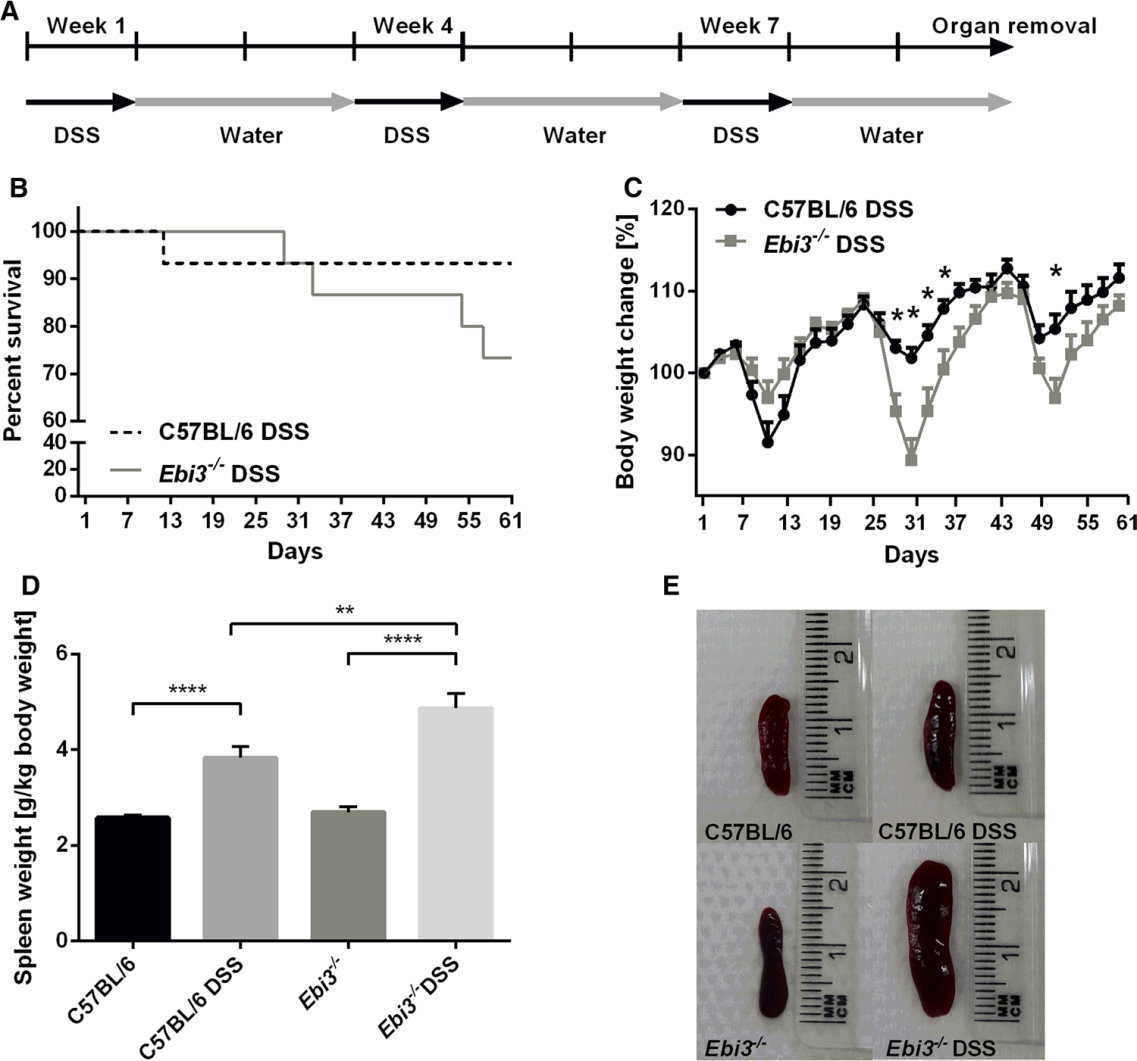
*Ebi3*^*−/−*^ mice are more sensitive towards chronic DSS-induced colitis than the wild-type. **a** The timeline represents the protocol for DSS-induced chronic colitis in C57BL/6 and *Ebi3*^*−/−*^ mice. **b** Effect of DSS treatment on survival rates of C57BL/6 and *Ebi3*^*−/−*^. The graph shows Kaplan–Meier curves of 15 mice per group. Statistical analysis was performed using log-rank test. **c** Effect of DSS treatment on body weight loss of each 15 C57BL/6 and *Ebi3*^*−/−*^ mice. Statistical analysis was performed using two-way ANOVA and Sidak’s post hoc test. **d, e** Effect of DSS treatment on spleen weight (**d**) and size (**e**) of C57BL/6 and *Ebi3*^*−/−*^. **d** The bars show the mean ± SEM from spleen weight of C57BL/6 and *Ebi3*^*−/−*^ treated with or without DSS. Statistical analysis was performed using one-way ANOVA and Tukey’s post hoc test (C57BL/6 DSS: *n* = 13; *Ebi3*^*−/−*^ DSS: *n* = 10; untreated C57BL/6: *n* = 15; untreated *Ebi3*^*−/−*^: *n* = 15, **p < 0.01; ****p < 0.0001). **e** The photographs illustrate the effect of DSS treatment on spleen size of C57BL/6 and *Ebi3*^*−/−*^ mice

As the two possible IL-12 family heterodimers of EBI3, IL-35 and IL-39, exert different effects, the in vivo impact of HDACi on colitis was of interest. First, a chronic DSS colitis model was induced in both, *Ebi3*^*−/−*^ mice and the related C57BL/6 wild-type mice; three cycles consisting of 7-day application of DSS to drinking water followed by a washout phase of 14 days (Fig. 3a). A dose of 1.5% DSS (w/v) for both strains was determined in advance as the most suitable compared to the dose of 1% DSS within a pilot project (unpublished). Comparison of colitis manifestations revealed that *Ebi3*^*−/−*^ mice developed more severe colitis than C57BL/6, as measured by survival (Fig. 3b), body weight course (Fig. 3c), and spleen weight and size (Fig. 3d, e). One wild-type and four of the *Ebi3*^*−/−*^ mice had to be sacrificed ahead of schedule, because the maximum acceptable body weight loss was reached resulting in mortality rates of 7% in wild-type and 27% in *EBI3*^*−/−*^ mice, respectively (Fig. 3b). In contrast to the knockout animals, wild-type mice recovered almost completely after DSS washout at the end of each treatment cycle. *Ebi3* deficiency associated with more pronounced negative impacts during inflammatory episodes of colitis progression, as assessed by body weight loss after the first cycle (Fig. 3c). Furthermore, spleen weight and size as common clinical parameters for inflammation were significantly increased in colitic *Ebi3*^*−/−*^ (Fig. 3d, e).

### The HDAC inhibitor SAHA improves colitis in wild-type but not in *Ebi3*^*−/−*^ mice

**Fig. 4:**
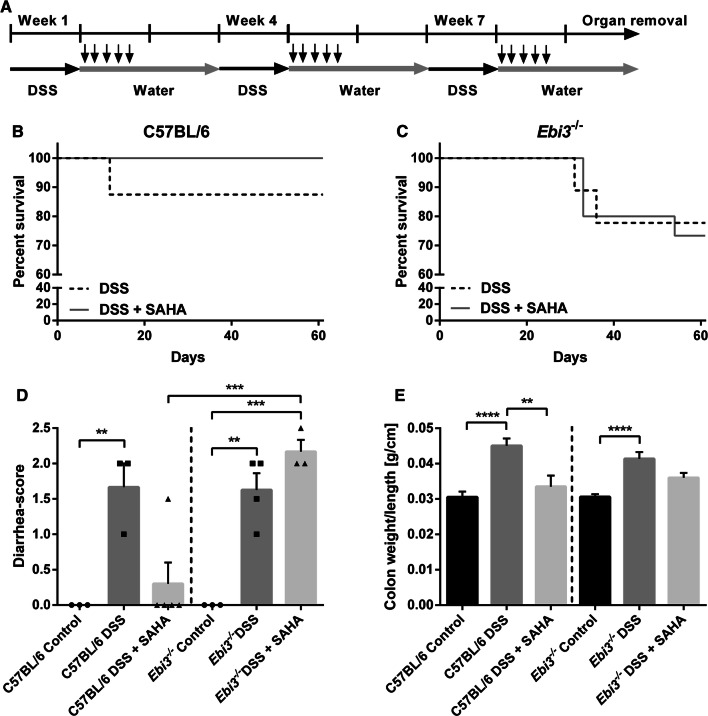
SAHA is only able to improve clinical parameters of colitis in the wild-type mice, not in *Ebi3*^*−/−*^. **a** The schedule visualizes the treatment regime for DSS and SAHA administration to C57BL/6 and *Ebi3*^*−/−*^. After each week of DSS treatment, C57BL/6 and *Ebi3*^*−/−*^ mice were treated daily for 5 days with SAHA or vehicle (i.p.) (administration indicated by arrows). **b**, **c** Influence of SAHA on survival rates of C57BL/6 (**b**) and *Ebi3*^*−/−*^ mice (**c**) compared to DSS-treated vehicle controls. The graphs show Kaplan–Meier curves of 15 mice per group. Statistical analysis was performed using log-rank test. **d, e** For the clinical evaluation of colitis, the stool consistency (**d**) and colon weight/length ratio (**e**) were determined. **d** Stool consistency was assessed per cage. The graph depicts the diarrhea-score of control mice and after DSS-induced colitis in C57BL/6 and *Ebi3*^*−/−*^ mice with or without SAHA treatment (untreated C57BL/6: *n* = 3; C57BL/6 DSS: *n* = 3; C57BL/6 DSS + SAHA: *n* = 5; untreated *Ebi3*^*−/−*^: *n* = 3; *Ebi3*^*−/−*^ DSS: *n* = 4; *Ebi3*^*−/−*^ DSS + SAHA: *n* = 5). Statistical analysis was performed using one-way ANOVA and Tukey’s post hoc test (**p < 0.01; ***p < 0.001). **e** Colon weight-to-length ratio (mean ± SEM) of control mice and after DSS-induced colitis in C57BL/6 and *Ebi3*^*−/−*^ mice with or without SAHA treatment (untreated C57BL/6: *n* = 5; C57BL/6 DSS: *n* = 5; C57BL/6 DSS + SAHA: *n* = 5; untreated *Ebi3*^*−/−*^: *n* = 15; *Ebi3*^*−/−*^ DSS: *n* = 7; *Ebi3*^*−/−*^ DSS + SAHA: *n* = 5). Statistical analysis was performed using one-way ANOVA and Tukey’s post hoc test (**p < 0.01; ****p < 0.0001)

Since the components of the anti-inflammatory cytokine IL-35 were epigenetically upregulated in HCEC under inflammatory conditions upon application of HDACi, we aimed to examine whether the epigenetic upregulation of EBI3 can be linked to the aforementioned observed amelioration of colitis by HDACi [19–21]. As before, colitis was induced in *Ebi3*^*−/−*^ and C57BL/6 wild-type mice by three cycles of DSS treatment and additionally treated with SAHA, an HDACi already approved for clinical use (Fig. 4a). SAHA improved the survival rates of DSS-treated wild-type mice (Fig. 4b). One animal of the vehicle-treated group had to be sacrificed after 2 weeks of treatment due to a sudden 30% weight loss (Fig. 4b). By contrast, SAHA had no impact on the survival rates of the DSS-treated *Ebi3*^*−/−*^ mice (Fig. 4c). To assess the extent of colitis, stool consistency and intestinal bleeding were determined. SAHA led to complete recovery from diarrhea at the end of the last cycle in C57BL/6 but not *Ebi3*^*−/−*^ mice (Fig. 4d).

As further parameters of colitis, colon length, and weight were determined at the end of the experiment. Compared to untreated controls, DSS significantly increased colon weight-to-length ratio in either mouse strain (Fig. 4e). Treatment with SAHA led to a significant reduction in the colonic weight-to-length ratio of DSS-treated wild-type mice but not DSS-treated *Ebi3*^−/−^ mice.

**Fig. 5:**
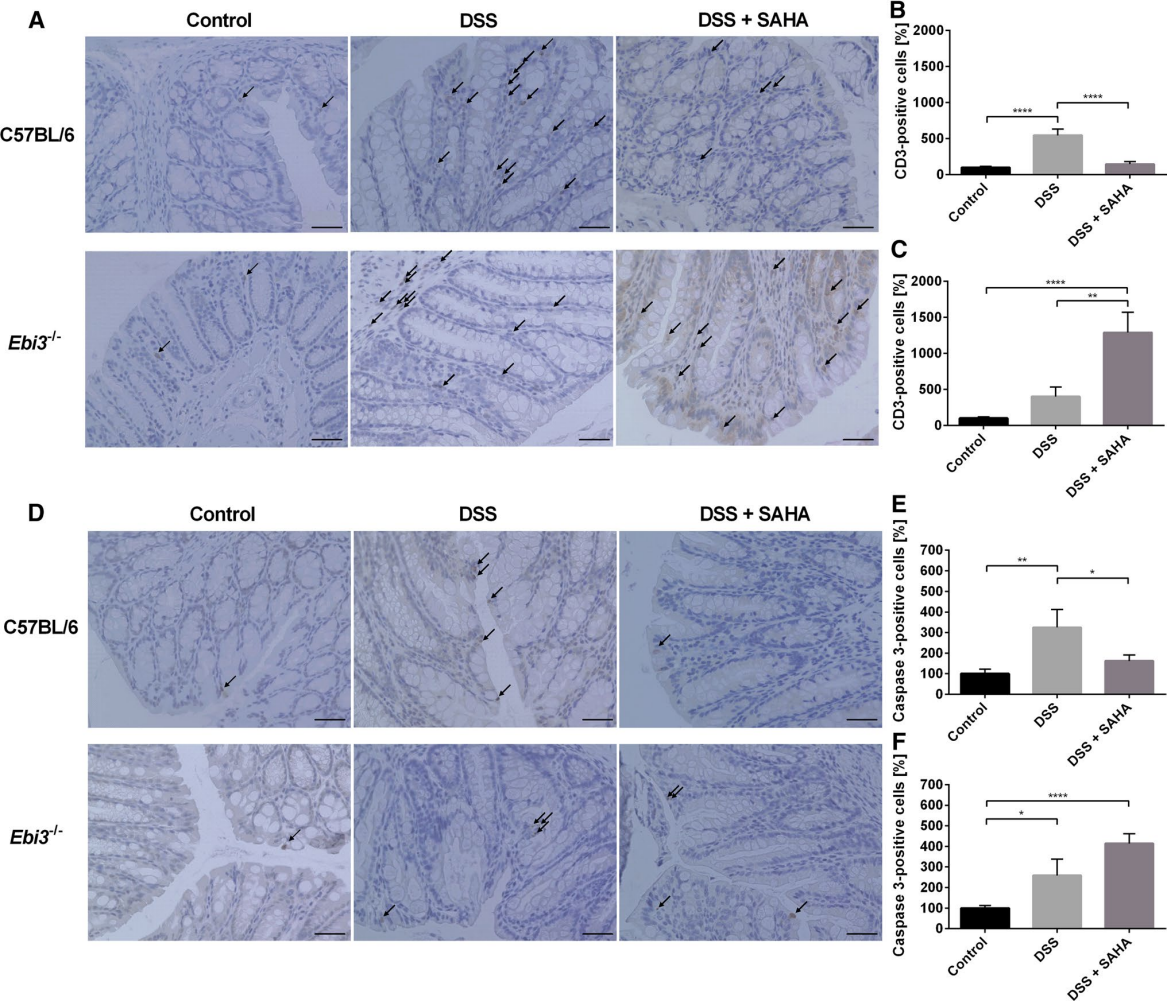
SAHA reduces the number of CD3- and Caspase-3-positive cells only in the wild-type mice. **a, d**. Cross-sections of formalin-fixed and paraffin-embedded colon tissue were prepared and stained with anti-CD3 (**a**) and anti-Caspase-3 (**d**) antibodies. The arrows indicate antigen positive cells. Scale bars: 40 µm. **b**, **c** Percentage of CD3-positive cells in C57BL/6 (**b**) (untreated: *n* = 15; DSS: *n* = 7; DSS + SAHA: *n* = 14) and *Ebi3*^*−/−*^ mice (**c**) (untreated: *n* = 15; DSS: *n* = 7; DSS + SAHA: *n* = 10). Statistical analysis was performed using one-way ANOVA and Tukey’s post hoc test. Data are shown as mean ± SEM (**p < 0.01; ****p < 0.0001). **e**, **f** Percentage of Caspase3-positive cells in C57BL/6 (**e**) (untreated: *n* = 15; DSS: *n* = 7; DSS + SAHA: *n* = 13) and *Ebi3*^*−/−*^ mice (**f**) (untreated: *n* = 15; DSS: *n* = 7; DSS + SAHA: *n* = 10). Statistical analysis was performed using one-way ANOVA and Tukey’s post hoc test. Data are shown as mean ± SEM (*p < 0.05; **p < 0.01; ****p < 0.0001)

For histological evaluation of colitis, colonic cross sections were examined immunohistochemically using anti-CD3 antibody as a T-cell marker (Fig. 5a–c). SAHA decreased the number of CD3-positive T lymphocytes in DSS-treated wild-type mice to untreated levels (Fig. 5b). In agreement with wild-type mice, DSS stimulation increased the number of CD3-positive inflammatory cells in *Ebi3*^*−/−*^. However, in contrast to the wild-type, SAHA was not able to decrease inflammatory cell invasion in *Ebi3*^*−/−*^. In fact, SAHA significantly increased the number of CD3-positive cells by more than threefold in the *Ebi3*-deficient animals (Fig. 5c). In addition to CD3, the sections were also stained with an antibody against Caspase-3, a marker of apoptosis (Fig. 5d–f). In both strains, Caspase 3 labeling was significantly increased upon DSS treatment. In wild-type mice, treatment with SAHA resulted in a 50% reduction of Caspase-3-positive cells, compared to the DSS-treated group (Fig. 5e). In *Ebi3*^*−/−*^, SAHA increased the number of Caspase-3-positive cells by 154% compared to *Ebi3*^*−/−*^ treated with DSS; however, this result was statistically not significant (Fig. 5f). In summary, SAHA was only able to reduce the inflammation in the wild-type mice. By contrast, SAHA seemingly aggravated the disease phenotype in *Ebi3*^*−/−*^ mice. Taken together, the results presented here indicate a central role of epigenetic EBI3 regulation in colitis.

### Influence of Ebi3 on upstream and downstream inflammatory signaling pathways depending on treatment with DSS or SAHA

To receive more information about the function of Ebi3 in inflammatory signaling pathways, the mRNA expression of target genes was analyzed by RT-qPCR in colon tissue of both mouse strains treated with DSS or DSS + SAHA or untreated (Supplementary Fig. S3). For the NfkB inflammatory pathway, the expression of *Tnfα*, *Inhibitor of nuclear factor Kappa B kinase subunit beta* (*Ikbkb*), and *Nfkb1* were analyzed as possible upstream inducers of IL-35. *IL-17a*, *B-cell lymphoma-2* (*Bcl-2*), and *B-cell lymphoma-extra large* (*Bcl-xL*) were analyzed as STAT3 target genes and *Interferon gamma* (*Ifnγ*) as a STAT4 target to discriminate between IL-35 and IL-39 signaling. Furthermore, *IL-10* and *IL-6* were tested as anti- and pro-inflammatory cytokines, respectively.

Comparing the treatment groups, there were no significant differences between *Ebi3*^*−/−*^ and C57BL/6 for *Tnfα* (Fig. S3a), *Ikbkb* (Fig. S3b), and *Nfkb1* (Fig. S3c), belonging to the inflammatory NfkB signaling pathway. Likewise, an altered gene expression of STAT3-mediated target genes *IL-17a* (Fig. S3d), *Bcl-2* (Fig. S3e), and *Bcl-xL* (Fig. S3f) was not detectable between *Ebi3*^*−/−*^ and C57BL/6 mice. Most interestingly, the STAT4 target *Ifnγ* mRNA expression (Fig. S3g) was significantly increased to more than 300% after DSS + SAHA treatment in C57BL/6. In contrast, this effect was almost abolished in *Ebi3*^*−/−*^, where *Ifnγ* mRNA levels were significantly reduced in all treatment groups compared to wild-type mice. The mRNA expression of the IL-35 target *IL-10* (Fig. S3h) was as well significantly increased in DSS-treated C57BL/6 compared to untreated controls. This upregulation did not occur in DSS-treated *Ebi3*^*−/−*^. On the other hand, the pro-inflammatory *IL-6* expression (Fig. S3i) was not significantly different between *Ebi3*^*−/−*^ and C57BL/6 for each treatment group.

## Discussion

The variability of the IL-12 cytokine family, mediated by the interchange of the subunits that make up their heterodimers, enables flexible adaptation to inflammatory processes that might play a role in the course of diseases like allergic rhinitis, asthma, and inflammatory bowel disease. While EBI3, as a component of the suppressive cytokines IL-35 and IL-27, is upregulated in acute inflammation, its expression decreases in chronic inflammatory diseases like psoriasis [30], allergic asthma [31], and allergic rhinitis [32]. Here, we aimed to examine the assumed epigenetic regulation of *EBI3 *in vitro and in vivo.

While the expression of IL-35 was previously shown in carcinoma cell lines [28], healthy tissue from liver, lung, kidney, and heart do not express *EBI3* [33], and, thus, are unable to form IL-35. Upregulation of IL-35 was seen in the acute phase of ulcerative colitis where it elicits anti-inflammatory effects [8]. This is consistent with our data, demonstrating that a cell line generated from normal human colon epithelium, HCEC, exhibits low-level basal expression of *EBI3* that could be increased by inflammatory triggers. In addition, we show for the first time that *EBI3* expression in HCECs is upregulated by epigenetic histone acetylation, as demonstrated by the application of two different HDACi, TSA as well as SAHA. Indeed, the actions of these HDACi were seen to greatly enhance *EBI3* induction by an inflammatory trigger, TNFα, in an apparently synergistically manner.

The molecular mechanisms underlying this synergistic effect are likely highly complex. On one hand, epigenetically active substances like TSA or SAHA inhibit histone deacetylases by directly blocking the active sites of the enzymes. As a result histones acetylation is preserved, which in turn elicits a relaxed euchromatin structure that facilitates gene transcription. TNFα, on the other hand, induces the nuclear translocation of the transcription factor NFκB, which subsequently stimulates gene expression of target genes by binding to their promoter regions to initiate RNA polymerase. In addition, there is evidence that HDACi have a direct and indirect impact on NFκB and NFκB-mediated transcription. Besides regulating the NFκB-dependent gene accessibility, acetylation events can prolong the inhibitor of kappa B kinase (IKK) activity, and NFκB protein subunits are directly acetylated resulting in transcriptional activation or increased binding to DNA [34]. However, our results from the in vivo studies do not show differential gene expression of the NFκB signaling mediators between C57BL/6 and *Ebi3*^*−/−*^. This is in line with the fact that EBI3 expression occurs downstream of the NFκB signaling and is upregulated as a reaction on acute inflammation to form the anti-inflammatory IL-35.

The increased expression of EBI3 by HDACi under inflammatory conditions should enable the increased formation of the anti-inflammatory cytokine IL-35 in HCEC, owing to the notable basal expression of the other IL-35 subunit, *IL-12p35,* a gene whose expression was also enhanced by HDACi and TNFα treatment. Although IL-35 was reported to be predominantly expressed in regulatory T lymphocytes [35], local expression of IL-35 in colon epithelium might help to restrict colitis. Due to the low *IL-12p40* levels in HCEC, only minor formation of IL-12 (IL-12p35/IL-12p40) and IL-23 (IL-23p19/IL-12p40) should be possible. And since the expression of *IL-27p28*, another binding partner of EBI3, is all but absent, the formation of IL-27 would be negligible. A further putative-binding partner of EBI3 might be the gene product of *IL-23p19* [11], which was highly expressed in HCEC. This heterodimer was suggested to be IL-39 and to play a role in neutrophil expansion in mice [11, 12, 31]. However, recent data doubt the formation of functional IL-39 in human beings [36, 37].

To further unravel the role of epigenetic EBI3 regulation in colitis, *Ebi3*^*−/−*^ mice incapable of IL-35 or IL-39 production were subjected to experimental DSS colitis. Compared to C57BL/6 mice, *Ebi3*^*−/−*^ mice exhibited more severe colitis symptoms, body weight loss, increased spleen weight, and lower survival rates. This suggests that in wild-type mice, the EBI3 subunit might preferentially dimerize with the IL-12p35 subunit to form the anti-inflammatory cytokine IL-35. Although attempts were made to clarify the binding partner of EBI3 by co-immunoprecipitation, this proved unfeasible owing to the near identical sizes of IL-12 family members and the known lack of reliable commercial antibodies [38, 39].

Therefore, we additionally performed mRNA expression analysis of downstream target genes to differentiate between IL-35 and IL-39 formation. Indeed, IL-35 target genes are activated by STAT1/STAT4 signaling [40], whereas IL-39 signals through STAT1/STAT3 [41]. Therefore, we analyzed *Ifnγ* as a STAT4 and *IL-17a*, *Bcl-2* and *Bcl-xL* as STAT3 target genes. While STAT4 target gene *Ifnγ* was differently expressed in treated *Ebi3*^−/−^ compared to wild-type, STAT3 target genes *IL-17a*, *Bcl-2,* and *Bcl-xL* were not significantly influenced by the *Ebi3* status. These findings underline that rather IL-35 than IL-39 is built in the applied mouse model of chronic colitis. Furthermore, this is in line with an upregulation of the anti-inflammatory cytokine *IL-10* in colitic wild-type mice compared to DSS-treated *Ebi3*^−/−^. An increase of IL-10 by IL-35 treatment was already described in an acute DSS-induced colitis mouse model [42] as well as in peripheral blood mononuclear cells from UC patients [17]. In conclusion, *IL-10* which is known to be upregulated by IL-35 was not induced in *Ebi3*^*−/−*^ mice, whereas *IL-6* seems to be independent of IL-35 formation.

Since IL-35 is able to restrict colitis symptoms, the epigenetic inducibility of EBI3 provides an eminently suitable target for medical intervention to counteract excessive inflammation during relapsing episodes of colitis. Regarding the HDACi used in this study, TSA stimulated *EBI3* expression in HCEC to a higher extent than SAHA. TSA is known to potently inhibit class I and II HDACs, but is not used therapeutically due to its pronounced toxicity. As such, it was not used in the animal experiments. SAHA likewise inhibits class I and II HDACs and is approved for the therapy of advanced refractory cutaneous T-cell lymphoma (CTCL). The fact that SAHA has a positive effect on colitis development has already been described [19–21], but the mechanism is still not well known. Our data demonstrate that SAHA-mediated elevation of IL-35 plays a role in this amelioration which was ascertained by treating *Ebi3*^*−/−*^ and the corresponding wild-type with SAHA and by comparing colitis manifestation in both strains. Colitis was more distinct in *Ebi3*^*−/−*^ compared with C57BL/6 wild-type mice, as seen by more pronounced loss of body weight, increase in spleen weight, and increased mortality. The clinically relevant HDACi SAHA significantly reduced several parameters of colitis in the wild-type mice. In particular, SAHA reduced T-lymphocyte infiltration, apoptosis, colon weight-to-length ratio, and improved the colitis symptoms and survival of wild-type mice. By contrast, SAHA appeared to aggravate the disease phenotype in *Ebi3*^−/−^ mice. In detail, survival, diarrhea, and the histopathologic markers CD3 and Caspase-3 were exacerbated.

In conclusion, histone deacetylation of *EBI3* plays a key role in colitis manifestation. Consistently, the data here suggest that histone acetylating conditions, such as upon SAHA application, improve colitis by a mechanism involving the local upregulation of *EBI3* and formation of the anti-inflammatory cytokine IL-35 in colon epithelium.

## Electronic supplementary material

Below is the link to the electronic supplementary material.

Supplementary file1 (PDF 711 kb)

## Abbreviations

Bcl-2: B-cell lymphoma-2
Bcl-xL: B-cell lymphoma-extra large
DMEM: Dulbecco’s modified eagle’s medium
DMSO: Dimethyl sulfoxide
DSS: Dextran sulfate sodium
Ebi3: Epstein–Barr virus-induced gene 3
HCEC: Human Colon Epithelial Cells
HDAC: Histone deacetylase
HDACi: Histone deacetylase inhibitor
HMBS: Hydroxymethylbilane synthase
Hprt: Hypoxanthine–guanine phosphoribosyltransferase
Ifnγ: Interferon gamma
Ikbkb: Inhibitor of nuclear factor Kappa B kinase subunit beta
IL: Interleukin
NfĸB: Nuclear factor ‘kappa-light-chain-enhancer’ of activated B-cells
RT-qPCR: Quantitative Reverse Transcription-PCR
SEM: Standard error of the mean
SDS-PAGE: SDS-polyacrylamide gel electrophoresis
STAT: Signal transducer and activator of transcription
TGF-β: Transforming growth factor ß
T_H_: Helper T cells
Treg: Regulatory T cells
TNFα: Tumor necrosis factor α
TSA: Trichostatin A
UC: Ulcerative colitis
