# Supplementary material for: Epigenetic histone modulation contributes to improvements in inflammatory bowel disease via EBI3
Source: Cell Mol Life Sci. 2020 Jan 18;77(23):5017–30. doi: 10.1007/s00018-020-03451-9 (PMC7658076; doi:10.1007/s00018-020-03451-9)
Supplement: Supplementary file 1 — Supplementary file1 (PDF 711 kb) [file 18_2020_3451_MOESM1_ESM.pdf]

Epigenetic histone modulation contributes to improvements in inflammatory bowel disease via EBI3

Cellular and Molecular Life Sciences

Alexandra Wetzel [1,#], Bettina Scholtka [1,#], Christian Gerecke [1], Burkhard Kleuser [1]

[1] University of Potsdam, Institute of Nutritional Science, Department of Nutritional Toxicology, Nuthetal, Germany

[#] These authors contributed equally to the study

Corresponding Author: Burkhard Kleuser, e-mail: kleuser@uni-potsdam.de

Supplementary Figures

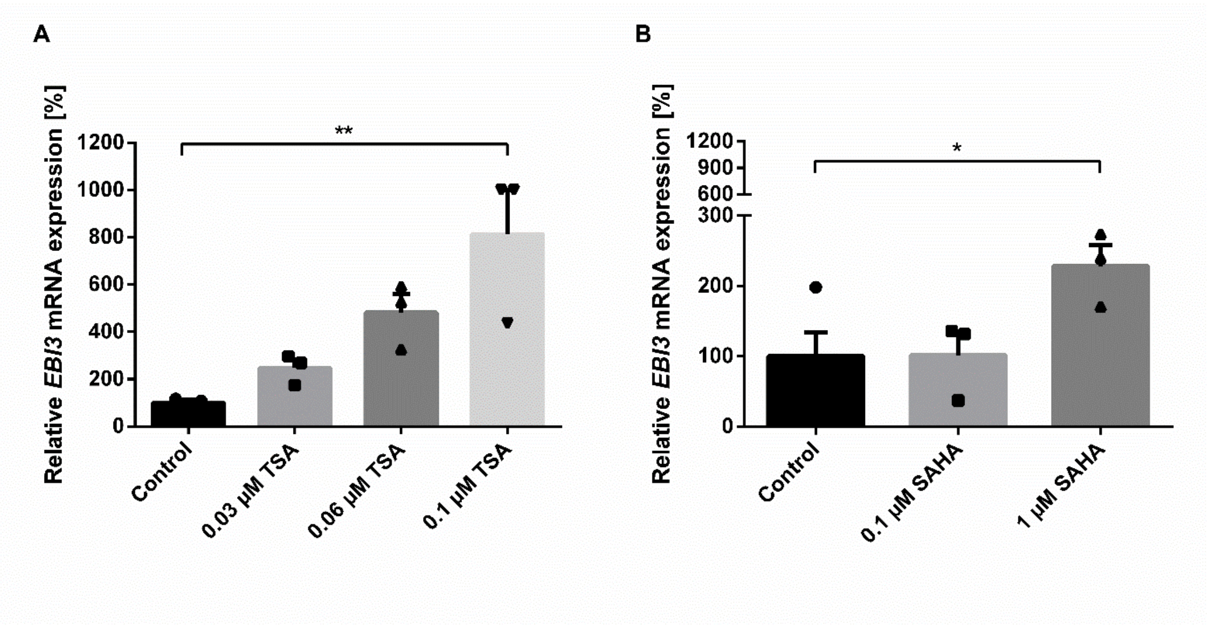

Supplementary Fig. S1: Concentration dependent *EBI3* mRNA expression in HCEC after treatment with HDACi. HCEC cells were stimulated with different concentrations of TSA (**a**) or SAHA (**b**) for 48 h. Cells treated with DMSO served as vehicle control. The mRNA expression of *EBI3* was determined by RT-qPCR and normalized to *HMBS* as housekeeping gene. **a** The graph shows the concentration dependent *EBI3* expression in HCEC by TSA (mean ± SEM from three independent experiments). Statistical analysis was performed using one-way ANOVA and Tukey's post-hoc test (**p <0.01). **b** HCEC exhibit concentration dependent *EBI3* expression following stimulation with SAHA (mean ± SEM from three independent experiments). Statistical analysis was performed using one-way ANOVA and Tukey's post-hoc test (*p <0.05)

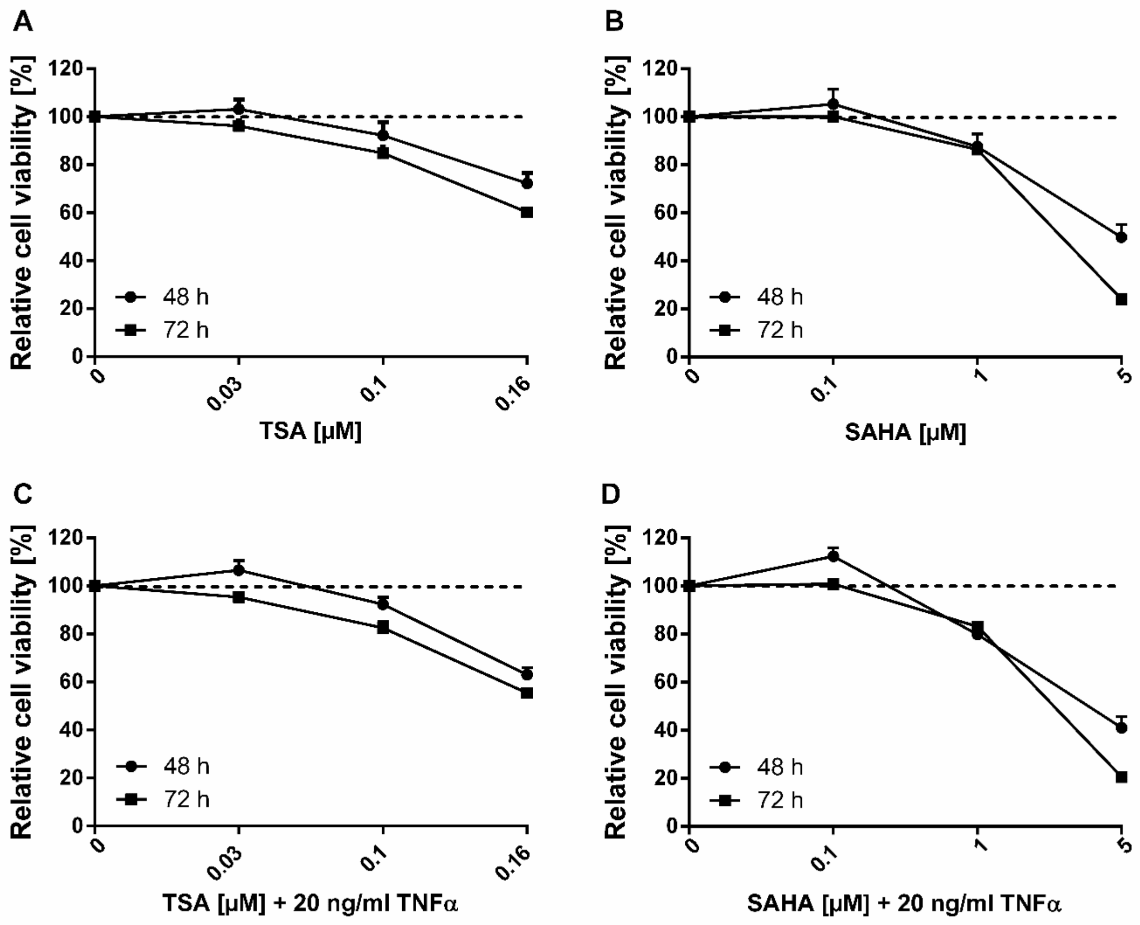

Supplementary Fig. S2: Effect of TNFα, TSA and SAHA on cell viability. HCEC cells were treated with the indicated concentrations of TSA (**a**) and SAHA (**b**) for 48 and 72 h. For the combinatorial stimulation, TNFα was added for the last 24 h (**c**, **d**). The cell viability was determined by MTT assay. The data are expressed as percentage of the untreated control and are presented as mean ± SEM from three independent experiments

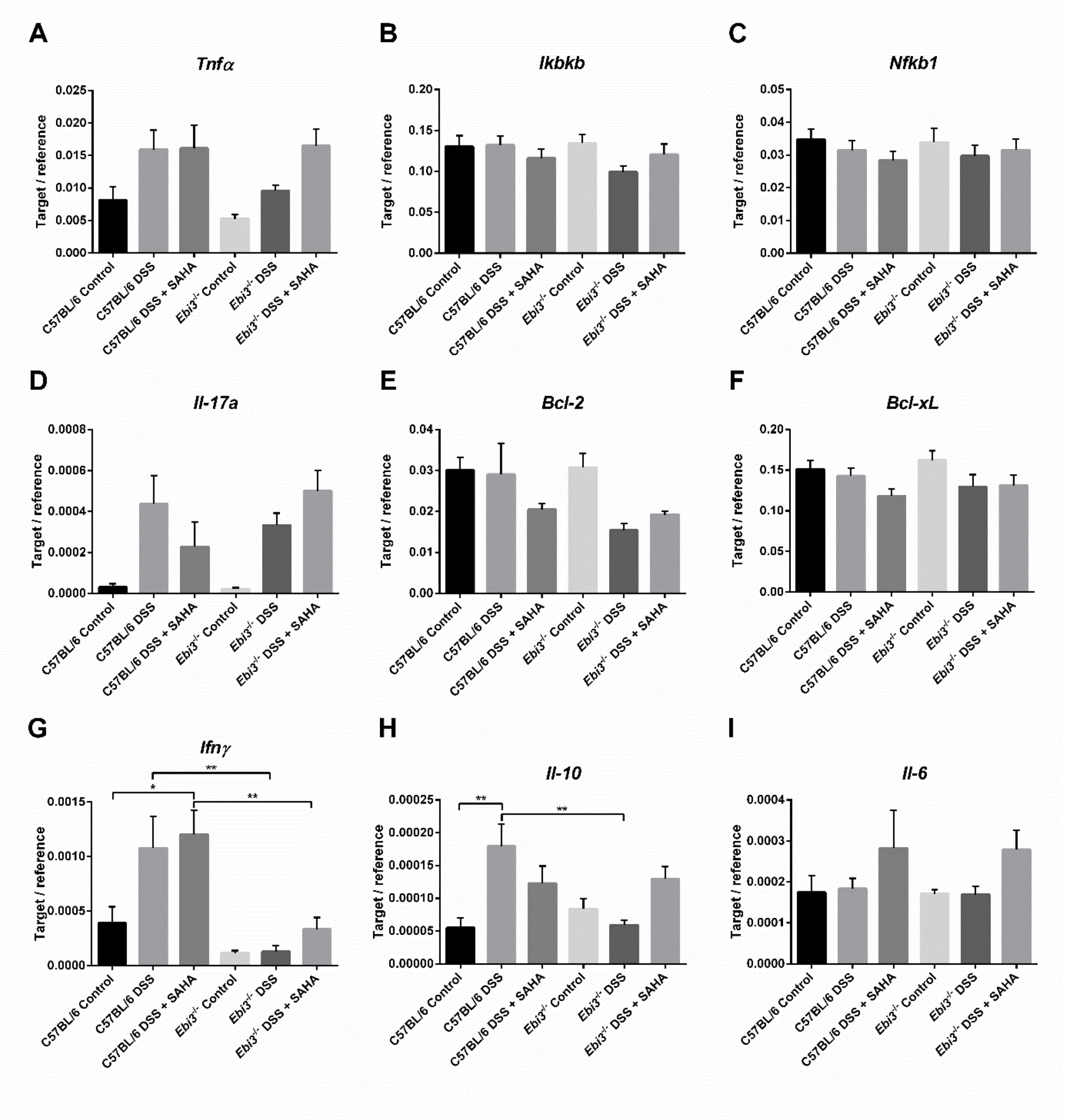

Supplementary Fig. S3: Effect of DSS and HDACi on the mRNA expressions of different NFκB and STAT-target genes. *Tnfα* (**a**), *Ikbkb* (**b**), *Nfkb1* (**c**), *IL-17a* (**d**), *Bcl-2* (**e**), *Bcl-xL* (**f**), *Ifnγ* (**g**), *IL-10* (**h**), and *IL-6* (**i**) mRNA expressions in murine colon tissues from C57BL/6 and *Ebi3*$^{-/-}$ mice were determined by RT-qPCR and normalized to *Hprt* as housekeeping gene. Data are presented as mean ± SEM from at least five animals (untreated C57BL/6: *n* = 6; C57BL/6 DSS: *n* = 7; C57BL/6 DSS + SAHA: *n* = 6; *Ebi3*$^{-/-}$ DSS: *n* = 6). Statistical analysis was performed using one-way ANOVA and Tukey's post-hoc test (*p <0.05; **p <0.01)
